# 4-(4-Chloro­phen­yl)-6-hydr­oxy-5-(2-thienyl­carbonyl)-6-(trifluoro­meth­yl)-3,4,5,6-tetra­hydro­pyrimidin-2(1*H*)-one monohydrate

**DOI:** 10.1107/S1600536809017097

**Published:** 2009-05-20

**Authors:** Mohammad Hossein Mosslemin, Mohammad Reza Nateghi, Hesamaddin Sadoughi, Asal Lamei

**Affiliations:** a Department of Chemistry, Islamic Azad University, Yazd Branch, Yazd, Iran

## Abstract

The asymmetric unit of the title compound, C_16_H_12_ClF_3_N_2_O_3_S·H_2_O, contains two crystallographically independent organic mol­ecules and two water mol­ecules. The organic species are linked by an inter­molecular O—H⋯O hydrogen bond, while the water mol­ecules are connected to them through inter­molecular O—H⋯N hydrogen bonds. The thio­phene and phenyl rings are oriented at dihedral angles of 62.35 (4) in the first independent mol­ecule and 60.74 (5)° in the second, while the pyrimidine rings adopt twisted conformations in both molecules. Intra­molecular N—H⋯F inter­actions result in the formation of two five-membered rings having envelope conformations. In the crystal structure, further inter­molecular O—H⋯O and N—H⋯O hydrogen bonds link the mol­ecules into chains.

## Related literature

For related structures, see: Paraskar *et al.* (2003 ▶); Peng & Deng (2001 ▶). For bond-length data, see: Allen *et al.* (1987 ▶).
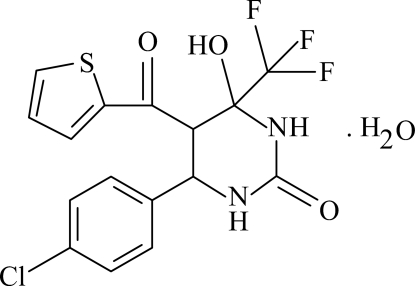


## Experimental

### 

#### Crystal data

C_16_H_12_ClF_3_N_2_O_3_S·H_2_O
*M* *_r_* = 422.81
Orthorhombic, 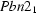

*a* = 9.1156 (6) Å
*b* = 14.1582 (7) Å
*c* = 27.6012 (17) Å
*V* = 3562.2 (4) Å^3^
*Z* = 8
Mo *K*α radiation
μ = 0.39 mm^−1^
*T* = 298 K
0.5 × 0.3 × 0.2 mm

#### Data collection

Bruker SMART CCD area-detector diffractometer
Absorption correction: multi-scan (*SADABS*; Sheldrick, 1998 ▶) *T*_min_ = 0.860, *T*_max_ = 0.923
19595 measured reflections
9251 independent reflections
7478 reflections with *I* > 2σ(*I*)
*R*_int_ = 0.032

#### Refinement

*R*[*F* ^2^ > 2σ(*F* ^2^)] = 0.055
*wR*(*F* ^2^) = 0.146
*S* = 1.07
9251 reflections
511 parameters
H atoms treated by a mixture of independent and constrained refinement
Δρ_max_ = 0.41 e Å^−3^
Δρ_min_ = −0.47 e Å^−3^
Absolute structure: Flack (1983 ▶), 4326 Friedel pairs
Flack parameter: 0.18 (8)

### 

Data collection: *SMART* (Bruker, 1998 ▶); cell refinement: *SAINT* (Bruker, 1998 ▶); data reduction: *SAINT*; program(s) used to solve structure: *SHELXTL* (Sheldrick, 2008 ▶); program(s) used to refine structure: *SHELXTL*; molecular graphics: *ORTEP-3 for Windows* (Farrugia, 1997 ▶); software used to prepare material for publication: *WinGX* (Farrugia, 1999 ▶).

## Supplementary Material

Crystal structure: contains datablocks global, I. DOI: 10.1107/S1600536809017097/hk2682sup1.cif

Structure factors: contains datablocks I. DOI: 10.1107/S1600536809017097/hk2682Isup2.hkl

Additional supplementary materials:  crystallographic information; 3D view; checkCIF report

## Figures and Tables

**Table 1:** Hydrogen-bond geometry (Å, °)

*D*—H⋯*A*	*D*—H	H⋯*A*	*D*⋯*A*	*D*—H⋯*A*
N1—H1*A*⋯F3	0.86	2.38	2.719 (4)	104
N1—H1*A*⋯O7^i^	0.86	2.37	3.022 (4)	132
N2—H2*A*⋯O7	0.86	2.15	2.914 (4)	147
O2—H2*B*⋯O6^ii^	0.89 (4)	1.85 (4)	2.694 (4)	156 (3)
N3—H3*A*⋯F4	0.86	2.41	2.733 (4)	103
N3—H3*A*⋯O8^iii^	0.86	2.34	3.007 (4)	134
N4—H4*A*⋯O8	0.86	2.14	2.921 (4)	151
O5—H5⋯O3	0.98 (6)	1.72 (6)	2.686 (4)	169 (6)
O7—H7*A*⋯O6^iii^	0.84 (7)	2.27 (7)	2.879 (4)	130 (5)
O7—H7*B*⋯O4^iv^	0.86 (6)	2.36 (6)	2.996 (5)	131 (5)
O7—H7*B*⋯O5^iv^	0.86 (6)	2.18 (6)	2.859 (4)	135 (5)
O8—H8*A*⋯O3^v^	0.75 (5)	2.21 (5)	2.897 (4)	153 (4)
O8—H8*B*⋯O1^i^	0.83 (5)	2.22 (6)	2.968 (5)	150 (5)
O8—H8*B*⋯O2^i^	0.83 (5)	2.27 (6)	2.861 (4)	128 (5)

Symmetry codes: (i) 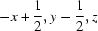; (ii) 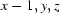; (iii) 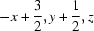; (iv) 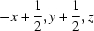; (v) 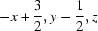.

**Fig. 1.**
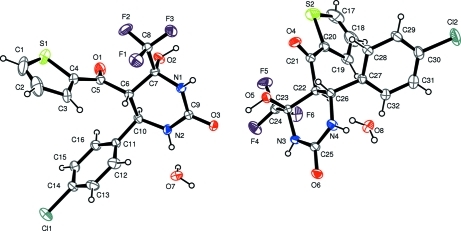
The molecular structure of the title molecule, with the atom-numbering scheme. Displacement ellipsoids are drawn at the 50% probability level.

**Fig. 2.**
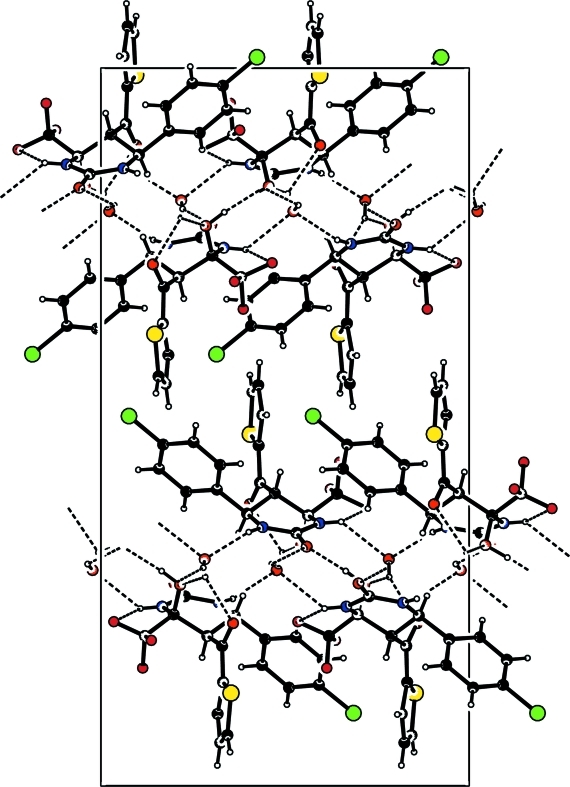
A partial packing diagram of the title compound viewed down the a axis. Hydrogen bonds are shown as dashed lines.

## supplementary crystallographic information

### Comment

In recent years, several modified and improved procedures for one-pot synthesis of dihydropyrimidine-2(1*H*)-ones have been reported (Paraskar *et al.*, 2003; Peng & Deng, 2001). However, in spite of their potential utility, many of these methods suffer from drawback like longer reaction times, unsatisfactory yields and cumbersome product isolation procedures. We report herein the synthesis and crystal structure of the title compound.

The asymmetric unit of the title compound contains two crystallographically independent molecules and two water molecules (Fig. 1 ), in which the bond lengths (Allen *et al.*,  1987) and angles are within normal ranges. Rings A (S1/C1–C4), C (C11–C16) and D (S2/C17–C20), F (C27–C32) are, of course, planar and they are oriented at dihedral angles of A/C = 62.35 (4)° and D/F = 60.74 (5)°. Rings B (N1/N2/C6/C7/C9/C10) and E (N3/N4/C22/C23/C25/C26) adopt twisted conformations. Intramolecular N—H···F interactions (Table 1) result in the formations of two five-membered rings G (N1/F3/C7/C8/H1A) and H (N3/F4/C23/C24/H3A) having envelope conformations with atoms C8 and C24 displaced by 0.594 (4) and -0.603 (5) Å from the planes of the other rings atoms. Intramolecular O—H···O hydrogen bond (Table 1) link the two molecules, while the water molecules are connected to them through the intramolecular N—H···O hydrogen bonds (Table 1).

In the crystal structure, intermolecular O—H···O and N—H···O hydrogen bonds (Table 1) link the molecules into chains (Fig. 2), in which they may be effective in the stabilization of the structure.

### Experimental

For the preparation of the title compound, a mixture of 1-(2-thenoyl)-3,3,3-trifluoroacetone (0.222 g, 1 mmol), 4-chlorobenzaldehyde (0.141 g, 1 mmol), urea (0.18 g, 3 mmol) and ammonium chloride (0.005 g, 0.1 mmol) were heated at 373 K under stirring for 20 min. After cooling, the reaction mixture was poured onto crushed ice (20 g). The separated solid was filtered, washed with cold water (20 ml) and recrystallized from ethylacetate–hexane (1:3) to afford pure product (yield; 78%, 0.330 g).

### Refinement

H atoms of water molecules and OH groups were located in difference Fourier map and refined isotropically. The remaining H atoms were positioned geometrically with N—H = 0.86 Å (for NH) and C—H = 0.93 and 0.98 Å, for aromatic and methine H atoms, respectively, and constrained to ride on their parent atoms, with U_iso_(H) = 1.2U_eq_(C,N).

### Crystal data

**Table:**

C_16_H_12_ClF_3_N_2_O_3_S·H_2_O	*F*(000) = 1728
*M**_r_* = 422.81	*D*_x_ = 1.577 Mg m^−^^3^
Orthorhombic, *P**b**n*2_1_	Mo *K*α radiation, λ = 0.71073 Å
Hall symbol: P 2c -2ab	Cell parameters from 1879 reflections
*a* = 9.1156 (6) Å	θ = 2.4–29.2°
*b* = 14.1582 (7) Å	µ = 0.39 mm^−^^1^
*c* = 27.6012 (17) Å	*T* = 298 K
*V* = 3562.2 (4) Å^3^	Block, colourless
*Z* = 8	0.5 × 0.3 × 0.2 mm

### Data collection

**Table:**

Bruker SMART CCD area-detector diffractometer	7478 reflections with *I* > 2σ(*I*)
φ and ω scans	*R*_int_ = 0.032
Absorption correction: multi-scan (*SADABS*; Sheldrick, 1998)	θ_max_ = 29.2°, θ_min_ = 2.4°
*T*_min_ = 0.860, *T*_max_ = 0.923	*h* = −11→12
19595 measured reflections	*k* = −19→19
9251 independent reflections	*l* = −37→36

### Refinement

**Table:**

Refinement on *F*^2^	H atoms treated by a mixture of independent and constrained refinement
Least-squares matrix: full	*w* = 1/[σ^2^(*F*_o_^2^) + (0.0679*P*)^2^ + 1.8489*P*] where *P* = (*F*_o_^2^ + 2*F*_c_^2^)/3
*R*[*F*^2^ > 2σ(*F*^2^)] = 0.055	(Δ/σ)_max_ = 0.012
*wR*(*F*^2^) = 0.146	Δρ_max_ = 0.41 e Å^−^^3^
*S* = 1.07	Δρ_min_ = −0.47 e Å^−^^3^
9251 reflections	Absolute structure: Flack (1983), 4326 Friedel pairs
511 parameters	Flack parameter: 0.18 (8)

### Special details

**Table:**

Geometry. All e.s.d.'s (except the e.s.d. in the dihedral angle between two l.s. planes) are estimated using the full covariance matrix. The cell e.s.d.'s are taken into account individually in the estimation of e.s.d.'s in distances, angles and torsion angles; correlations between e.s.d.'s in cell parameters are only used when they are defined by crystal symmetry. An approximate (isotropic) treatment of cell e.s.d.'s is used for estimating e.s.d.'s involving l.s. planes.

### Fractional atomic coordinates and isotropic or equivalent isotropic displacement parameters (Å^2^)

**Table:**

	*x*	*y*	*z*	*U*_iso_*/*U*_eq_	
Cl1	0.20863 (18)	1.18591 (11)	0.10132 (6)	0.0800 (4)	
Cl2	0.71737 (17)	0.07620 (11)	0.51510 (6)	0.0829 (5)	
S1	−0.37400 (15)	0.85338 (12)	0.12927 (6)	0.0797 (4)	
S2	0.13318 (16)	0.40572 (12)	0.48988 (7)	0.0805 (4)	
O1	−0.2355 (3)	0.8588 (2)	0.22653 (14)	0.0540 (8)	
O2	−0.0969 (3)	0.71519 (19)	0.27993 (10)	0.0401 (6)	
H2B	−0.086 (4)	0.665 (3)	0.2992 (15)	0.028 (10)*	
O3	0.3549 (3)	0.69972 (17)	0.28201 (10)	0.0414 (6)	
O4	0.2597 (3)	0.4046 (3)	0.39238 (14)	0.0539 (8)	
O5	0.4027 (3)	0.54419 (17)	0.33543 (9)	0.0394 (5)	
H5	0.396 (7)	0.604 (4)	0.318 (2)	0.088 (18)*	
O6	0.8546 (3)	0.55885 (17)	0.33323 (10)	0.0413 (6)	
O7	0.3736 (3)	0.9773 (2)	0.30044 (11)	0.0433 (6)	
H7A	0.459 (8)	0.964 (4)	0.309 (2)	0.09 (2)*	
H7B	0.321 (7)	0.993 (4)	0.325 (2)	0.080 (18)*	
O8	0.8726 (3)	0.2819 (2)	0.31572 (11)	0.0423 (6)	
H8A	0.946 (6)	0.278 (3)	0.3038 (17)	0.045 (13)*	
H8B	0.820 (6)	0.283 (4)	0.291 (2)	0.066 (15)*	
N1	0.1334 (3)	0.66639 (19)	0.24968 (11)	0.0335 (6)	
H1A	0.1546	0.6074	0.247	0.04*	
N2	0.2184 (4)	0.8206 (2)	0.25512 (13)	0.0408 (7)	
H2A	0.291	0.8579	0.2605	0.049*	
N3	0.6328 (3)	0.59341 (18)	0.36587 (10)	0.0314 (5)	
H3A	0.6543	0.6524	0.3679	0.038*	
N4	0.7153 (4)	0.43923 (19)	0.36029 (13)	0.0385 (7)	
H4A	0.7864	0.4015	0.3535	0.046*	
F1	−0.0306 (3)	0.61345 (17)	0.16413 (9)	0.0574 (6)	
F2	−0.2295 (3)	0.6369 (2)	0.20268 (16)	0.0684 (9)	
F3	−0.0748 (3)	0.53514 (14)	0.22866 (10)	0.0553 (6)	
F4	0.4222 (3)	0.72493 (15)	0.38632 (10)	0.0572 (6)	
F5	0.2705 (3)	0.6231 (2)	0.41348 (15)	0.0686 (9)	
F6	0.4700 (3)	0.64863 (18)	0.45092 (9)	0.0618 (7)	
C1	−0.3412 (9)	0.8435 (4)	0.0706 (3)	0.094 (2)	
H1	−0.4133	0.8506	0.047	0.112*	
C2	−0.2010 (10)	0.8242 (5)	0.0601 (2)	0.084 (2)	
H2	−0.1682	0.8161	0.0285	0.1*	
C3	−0.1067 (5)	0.8169 (3)	0.10026 (15)	0.0440 (8)	
H3	−0.0063	0.8051	0.0994	0.053*	
C4	−0.1978 (4)	0.8318 (3)	0.14442 (15)	0.0426 (8)	
C5	−0.1541 (4)	0.8292 (2)	0.19488 (13)	0.0343 (7)	
C6	−0.0040 (3)	0.7890 (2)	0.20871 (12)	0.0286 (6)	
H6	0.0497	0.7749	0.1788	0.034*	
C7	−0.0144 (3)	0.6974 (2)	0.23879 (11)	0.0288 (6)	
C8	−0.0881 (4)	0.6194 (2)	0.20873 (13)	0.0366 (7)	
C9	0.2396 (3)	0.7281 (3)	0.26401 (15)	0.0311 (7)	
C10	0.0830 (4)	0.8633 (2)	0.23712 (12)	0.0314 (6)	
H10	0.0241	0.8855	0.2646	0.038*	
C11	0.1184 (3)	0.9461 (2)	0.20424 (12)	0.0319 (6)	
C12	0.2245 (5)	0.9376 (3)	0.1683 (2)	0.0462 (9)	
H12	0.2777	0.8818	0.1655	0.055*	
C13	0.2516 (4)	1.0109 (3)	0.13683 (18)	0.0466 (11)	
H13	0.3236	1.0052	0.1131	0.056*	
C14	0.1722 (4)	1.0921 (3)	0.14066 (14)	0.0453 (8)	
C15	0.0667 (5)	1.1025 (3)	0.17562 (15)	0.0461 (9)	
H15	0.0139	1.1586	0.178	0.055*	
C16	0.0395 (4)	1.0292 (2)	0.20716 (14)	0.0397 (7)	
H16	−0.0328	1.0358	0.2307	0.048*	
C17	0.1708 (9)	0.4116 (5)	0.5484 (3)	0.088 (2)	
H17	0.0997	0.4045	0.5723	0.105*	
C18	0.3112 (9)	0.4273 (4)	0.5583 (2)	0.0804 (18)	
H18	0.3463	0.4308	0.5899	0.096*	
C19	0.4056 (5)	0.4386 (3)	0.51657 (13)	0.0444 (8)	
H19	0.506	0.4501	0.5167	0.053*	
C20	0.3103 (4)	0.4283 (3)	0.47414 (15)	0.0421 (8)	
C21	0.3463 (4)	0.4322 (2)	0.42239 (14)	0.0365 (7)	
C22	0.4972 (3)	0.4722 (2)	0.40755 (11)	0.0287 (6)	
H22	0.5523	0.4871	0.4371	0.034*	
C23	0.4852 (3)	0.5625 (2)	0.37684 (11)	0.0285 (6)	
C24	0.4120 (4)	0.6412 (2)	0.40686 (14)	0.0386 (7)	
C25	0.7380 (4)	0.5309 (2)	0.35238 (16)	0.0331 (8)	
C26	0.5819 (3)	0.3969 (2)	0.37927 (12)	0.0307 (6)	
H26	0.5215	0.3742	0.3523	0.037*	
C27	0.6181 (3)	0.3148 (2)	0.41269 (12)	0.0307 (6)	
C28	0.5391 (4)	0.2319 (2)	0.40975 (14)	0.0401 (7)	
H28	0.4665	0.2253	0.3863	0.048*	
C29	0.5675 (4)	0.1575 (3)	0.44180 (17)	0.0499 (9)	
H29	0.5138	0.1017	0.4402	0.06*	
C30	0.6760 (4)	0.1689 (3)	0.47547 (15)	0.0492 (10)	
C31	0.7576 (5)	0.2514 (3)	0.4788 (2)	0.0515 (12)	
H31	0.8306	0.2575	0.5021	0.062*	
C32	0.7287 (4)	0.3237 (3)	0.44722 (16)	0.0398 (8)	
H32	0.7833	0.3791	0.4488	0.048*	

### Atomic displacement parameters (Å^2^)

**Table:**

	*U*^11^	*U*^22^	*U*^33^	*U*^12^	*U*^13^	*U*^23^
Cl1	0.0826 (8)	0.0777 (8)	0.0797 (9)	−0.0138 (7)	0.0002 (8)	0.0486 (7)
Cl2	0.0798 (8)	0.0833 (8)	0.0856 (10)	0.0318 (7)	0.0201 (8)	0.0568 (8)
S1	0.0514 (6)	0.0965 (10)	0.0912 (11)	0.0052 (7)	−0.0265 (7)	0.0229 (8)
S2	0.0571 (7)	0.0907 (10)	0.0937 (11)	−0.0052 (7)	0.0273 (8)	0.0203 (8)
O1	0.0466 (16)	0.0612 (18)	0.054 (2)	0.0213 (13)	0.0096 (13)	0.0121 (15)
O2	0.0436 (14)	0.0339 (12)	0.0427 (14)	0.0050 (11)	0.0152 (11)	0.0097 (11)
O3	0.0324 (12)	0.0356 (12)	0.0561 (16)	−0.0016 (9)	−0.0148 (11)	0.0126 (11)
O4	0.0398 (15)	0.0719 (19)	0.050 (2)	−0.0212 (13)	−0.0117 (12)	0.0137 (15)
O5	0.0443 (13)	0.0339 (12)	0.0400 (13)	−0.0061 (10)	−0.0126 (11)	0.0086 (10)
O6	0.0356 (12)	0.0367 (12)	0.0515 (15)	0.0034 (10)	0.0149 (11)	0.0112 (11)
O7	0.0327 (13)	0.0486 (15)	0.0486 (16)	−0.0070 (11)	0.0040 (11)	−0.0024 (12)
O8	0.0315 (13)	0.0482 (15)	0.0472 (15)	0.0060 (11)	−0.0037 (12)	−0.0004 (12)
N1	0.0298 (12)	0.0255 (12)	0.0451 (17)	0.0001 (10)	−0.0053 (11)	0.0014 (11)
N2	0.0395 (14)	0.0272 (13)	0.056 (2)	−0.0045 (12)	−0.0210 (15)	0.0089 (12)
N3	0.0281 (12)	0.0246 (11)	0.0414 (15)	−0.0004 (9)	0.0072 (11)	0.0021 (10)
N4	0.0399 (14)	0.0280 (12)	0.0475 (18)	0.0089 (12)	0.0166 (14)	0.0047 (11)
F1	0.0750 (16)	0.0529 (13)	0.0443 (13)	−0.0109 (12)	−0.0044 (12)	−0.0085 (11)
F2	0.0333 (11)	0.0598 (15)	0.112 (3)	−0.0091 (11)	−0.0239 (15)	0.0002 (16)
F3	0.0688 (15)	0.0297 (9)	0.0674 (16)	−0.0121 (10)	−0.0203 (13)	0.0055 (10)
F4	0.0739 (16)	0.0313 (10)	0.0664 (16)	0.0138 (10)	0.0233 (13)	0.0062 (10)
F5	0.0312 (10)	0.0600 (15)	0.115 (3)	0.0080 (10)	0.0226 (15)	−0.0060 (16)
F6	0.0785 (18)	0.0637 (14)	0.0433 (13)	0.0138 (13)	0.0042 (12)	−0.0161 (11)
C1	0.115 (5)	0.076 (4)	0.090 (5)	0.007 (4)	−0.071 (4)	0.007 (3)
C2	0.132 (6)	0.077 (4)	0.041 (3)	0.015 (4)	−0.022 (3)	0.003 (2)
C3	0.053 (2)	0.0481 (19)	0.0310 (17)	0.0122 (16)	−0.0079 (16)	0.0067 (15)
C4	0.0383 (18)	0.0404 (17)	0.049 (2)	0.0002 (14)	−0.0079 (15)	0.0121 (16)
C5	0.0302 (15)	0.0331 (15)	0.0397 (18)	0.0016 (12)	0.0018 (12)	0.0065 (13)
C6	0.0256 (13)	0.0301 (13)	0.0302 (15)	−0.0005 (10)	0.0013 (11)	0.0037 (12)
C7	0.0250 (13)	0.0266 (12)	0.0348 (15)	−0.0014 (10)	−0.0003 (11)	0.0006 (11)
C8	0.0340 (15)	0.0332 (15)	0.0427 (18)	−0.0031 (12)	−0.0074 (14)	0.0016 (13)
C9	0.0307 (16)	0.0329 (15)	0.0297 (19)	−0.0014 (12)	−0.0019 (11)	0.0048 (13)
C10	0.0381 (16)	0.0238 (12)	0.0322 (16)	0.0010 (11)	−0.0003 (12)	0.0029 (11)
C11	0.0342 (15)	0.0302 (14)	0.0312 (15)	−0.0020 (11)	−0.0042 (12)	0.0048 (12)
C12	0.0484 (19)	0.0333 (17)	0.057 (3)	0.0017 (15)	0.012 (2)	−0.0047 (16)
C13	0.051 (3)	0.051 (2)	0.037 (3)	−0.0155 (17)	0.0098 (15)	0.0014 (18)
C14	0.0478 (19)	0.0446 (19)	0.0435 (19)	−0.0134 (16)	−0.0069 (16)	0.0182 (16)
C15	0.047 (2)	0.0348 (16)	0.056 (2)	0.0060 (15)	−0.0049 (17)	0.0120 (16)
C16	0.0369 (16)	0.0360 (16)	0.0464 (19)	0.0024 (13)	0.0037 (15)	0.0045 (15)
C17	0.106 (5)	0.074 (4)	0.083 (4)	0.001 (3)	0.059 (4)	0.014 (3)
C18	0.122 (6)	0.070 (3)	0.050 (3)	0.0003 (3)	0.017 (3)	0.015 (2)
C19	0.054 (2)	0.052 (2)	0.0271 (17)	0.0350 (3)	0.0152 (15)	0.0050 (15)
C20	0.0339 (17)	0.0429 (18)	0.050 (2)	0.0030 (14)	0.0108 (15)	0.0132 (15)
C21	0.0302 (15)	0.0361 (15)	0.0431 (18)	−0.0016 (12)	0.0007 (13)	0.0118 (14)
C22	0.0261 (13)	0.0304 (13)	0.0297 (15)	−0.0015 (10)	−0.0009 (11)	0.0030 (11)
C23	0.0252 (13)	0.0282 (13)	0.0321 (15)	−0.0007 (10)	−0.0010 (11)	0.0027 (11)
C24	0.0289 (14)	0.0389 (16)	0.048 (2)	0.0046 (12)	0.0079 (14)	0.0032 (14)
C25	0.0289 (16)	0.0306 (15)	0.040 (2)	0.0029 (11)	0.0083 (12)	0.0042 (14)
C26	0.0335 (14)	0.0278 (13)	0.0309 (15)	−0.0023 (11)	0.0024 (12)	0.0058 (11)
C27	0.0295 (13)	0.0270 (13)	0.0355 (16)	0.0035 (11)	0.0015 (12)	0.0037 (12)
C28	0.0408 (16)	0.0333 (15)	0.046 (2)	−0.0028 (13)	0.0000 (15)	0.0101 (14)
C29	0.047 (2)	0.0364 (17)	0.067 (3)	−0.0020 (15)	0.0094 (19)	0.0194 (17)
C30	0.0469 (19)	0.052 (2)	0.049 (2)	0.0184 (17)	0.0188 (17)	0.0210 (18)
C31	0.053 (3)	0.055 (2)	0.046 (3)	0.0143 (18)	−0.0053 (17)	0.004 (2)
C32	0.0390 (17)	0.0401 (17)	0.040 (2)	0.0013 (14)	−0.0057 (15)	0.0058 (15)

### Geometric parameters (Å, °)

**Table:**

O2—H2B	0.89 (4)	C14—C15	1.370 (6)
O5—H5	0.99 (6)	C14—Cl1	1.747 (4)
O7—H7A	0.84 (7)	C15—C16	1.377 (5)
O7—H7B	0.87 (7)	C15—H15	0.93
O8—H8A	0.75 (5)	C16—H16	0.93
O8—H8B	0.84 (6)	C17—C18	1.328 (11)
N1—H1A	0.86	C17—S2	1.652 (8)
N2—H2A	0.86	C17—H17	0.93
N3—H3A	0.86	C18—C19	1.448 (7)
N4—H4A	0.86	C18—H18	0.93
C1—C2	1.338 (11)	C19—C20	1.465 (6)
C1—S1	1.653 (8)	C19—H19	0.93
C1—H1	0.93	C20—C21	1.467 (5)
C2—C3	1.407 (7)	C20—S2	1.703 (4)
C2—H2	0.93	C21—O4	1.209 (5)
C3—C4	1.490 (6)	C21—C22	1.542 (4)
C3—H3	0.93	C22—C26	1.530 (4)
C4—C5	1.449 (5)	C22—C23	1.538 (4)
C4—S1	1.688 (4)	C22—H22	0.98
C5—O1	1.221 (5)	C23—O5	1.393 (4)
C5—C6	1.530 (4)	C23—N3	1.447 (4)
C6—C10	1.533 (4)	C23—C24	1.541 (4)
C6—C7	1.543 (4)	C24—F4	1.317 (4)
C6—H6	0.98	C24—F5	1.328 (4)
C7—O2	1.385 (4)	C24—F6	1.330 (5)
C7—N1	1.448 (4)	C25—O6	1.252 (4)
C7—C8	1.536 (4)	C25—N4	1.332 (4)
C8—F3	1.319 (4)	C25—N3	1.357 (4)
C8—F2	1.323 (4)	C26—N4	1.453 (4)
C8—F1	1.340 (4)	C26—C27	1.520 (4)
C9—O3	1.230 (4)	C26—H26	0.98
C9—N2	1.347 (4)	C27—C28	1.380 (5)
C9—N1	1.363 (4)	C27—C32	1.393 (5)
C10—N2	1.461 (4)	C28—C29	1.399 (5)
C10—C11	1.517 (4)	C28—H28	0.93
C10—H10	0.98	C29—C30	1.367 (6)
C11—C16	1.382 (5)	C29—H29	0.93
C11—C12	1.391 (5)	C30—C31	1.388 (7)
C12—C13	1.376 (6)	C30—Cl2	1.750 (4)
C12—H12	0.93	C31—C32	1.370 (6)
C13—C14	1.363 (6)	C31—H31	0.93
C13—H13	0.93	C32—H32	0.93
			
C1—S1—C4	93.2 (3)	C13—C14—C15	121.2 (4)
C17—S2—C20	92.5 (3)	C13—C14—Cl1	119.5 (3)
C7—O2—H2B	107 (3)	C15—C14—Cl1	119.3 (3)
C23—O5—H5	106 (4)	C14—C15—C16	119.4 (3)
H7A—O7—H7B	110 (6)	C14—C15—H15	120.3
H8A—O8—H8B	99 (5)	C16—C15—H15	120.3
C7—N1—H1A	119.1	C15—C16—C11	120.8 (3)
C9—N1—C7	121.8 (3)	C15—C16—H16	119.6
C9—N1—H1A	119.1	C11—C16—H16	119.6
C9—N2—C10	125.9 (3)	C18—C17—S2	114.3 (4)
C9—N2—H2A	117.1	C18—C17—H17	122.9
C10—N2—H2A	117.1	S2—C17—H17	122.9
C23—N3—H3A	119.4	C17—C18—C19	115.2 (5)
C25—N3—C23	121.1 (3)	C17—C18—H18	122.4
C25—N3—H3A	119.4	C19—C18—H18	122.4
C25—N4—C26	126.2 (3)	C18—C19—C20	105.9 (4)
C25—N4—H4A	116.9	C18—C19—H19	127.1
C26—N4—H4A	116.9	C20—C19—H19	127.1
C2—C1—S1	113.7 (4)	C19—C20—C21	130.0 (3)
C2—C1—H1	123.2	C19—C20—S2	112.1 (3)
S1—C1—H1	123.2	C21—C20—S2	117.9 (3)
C1—C2—C3	115.4 (6)	O4—C21—C20	120.6 (3)
C1—C2—H2	122.3	O4—C21—C22	121.3 (3)
C3—C2—H2	122.3	C20—C21—C22	118.2 (3)
C2—C3—C4	107.1 (4)	C26—C22—C23	109.5 (2)
C2—C3—H3	126.5	C26—C22—C21	109.3 (2)
C4—C3—H3	126.5	C23—C22—C21	112.8 (2)
C5—C4—C3	129.0 (3)	C26—C22—H22	108.4
C5—C4—S1	120.3 (3)	C23—C22—H22	108.4
C3—C4—S1	110.7 (3)	C21—C22—H22	108.4
O1—C5—C4	120.8 (3)	O5—C23—N3	112.8 (3)
O1—C5—C6	119.5 (3)	O5—C23—C22	109.6 (2)
C4—C5—C6	119.7 (3)	N3—C23—C22	107.5 (2)
C5—C6—C10	109.6 (2)	O5—C23—C24	110.0 (3)
C5—C6—C7	113.1 (2)	N3—C23—C24	107.3 (2)
C10—C6—C7	109.5 (2)	C22—C23—C24	109.6 (2)
C5—C6—H6	108.2	F4—C24—F5	107.6 (3)
C10—C6—H6	108.2	F4—C24—F6	107.1 (3)
C7—C6—H6	108.2	F5—C24—F6	106.0 (3)
O2—C7—N1	112.9 (3)	F4—C24—C23	112.9 (3)
O2—C7—C8	109.6 (3)	F5—C24—C23	110.8 (3)
N1—C7—C8	107.5 (2)	F6—C24—C23	112.1 (3)
O2—C7—C6	108.8 (2)	O6—C25—N4	120.6 (3)
N1—C7—C6	108.0 (2)	O6—C25—N3	120.6 (3)
C8—C7—C6	109.9 (2)	N4—C25—N3	118.8 (3)
F3—C8—F2	108.2 (3)	N4—C26—C27	110.6 (3)
F3—C8—F1	106.9 (3)	N4—C26—C22	108.6 (2)
F2—C8—F1	106.0 (3)	C27—C26—C22	109.4 (3)
F3—C8—C7	112.7 (3)	N4—C26—H26	109.4
F2—C8—C7	111.0 (3)	C27—C26—H26	109.4
F1—C8—C7	111.7 (3)	C22—C26—H26	109.4
O3—C9—N2	120.9 (3)	C28—C27—C32	119.6 (3)
O3—C9—N1	121.0 (3)	C28—C27—C26	120.1 (3)
N2—C9—N1	118.0 (3)	C32—C27—C26	120.2 (3)
N2—C10—C11	110.1 (3)	C27—C28—C29	120.5 (4)
N2—C10—C6	109.1 (2)	C27—C28—H28	119.8
C11—C10—C6	109.5 (3)	C29—C28—H28	119.8
N2—C10—H10	109.4	C30—C29—C28	118.3 (4)
C11—C10—H10	109.4	C30—C29—H29	120.8
C6—C10—H10	109.4	C28—C29—H29	120.8
C16—C11—C12	118.5 (3)	C29—C30—C31	122.2 (4)
C16—C11—C10	120.8 (3)	C29—C30—Cl2	119.5 (3)
C12—C11—C10	120.5 (3)	C31—C30—Cl2	118.3 (4)
C13—C12—C11	120.6 (4)	C32—C31—C30	118.9 (4)
C13—C12—H12	119.7	C32—C31—H31	120.6
C11—C12—H12	119.7	C30—C31—H31	120.6
C14—C13—C12	119.5 (4)	C31—C32—C27	120.5 (4)
C14—C13—H13	120.3	C31—C32—H32	119.7
C12—C13—H13	120.3	C27—C32—H32	119.7
			
S1—C1—C2—C3	−0.8 (8)	C26—C22—C23—O5	64.1 (3)
C1—C2—C3—C4	1.3 (7)	C21—C22—C23—O5	−57.8 (3)
C2—C3—C4—C5	178.4 (5)	C26—C22—C23—N3	−58.8 (3)
C2—C3—C4—S1	−1.2 (5)	C21—C22—C23—N3	179.3 (3)
C3—C4—C5—O1	167.1 (4)	C26—C22—C23—C24	−175.1 (3)
S1—C4—C5—O1	−13.2 (5)	C21—C22—C23—C24	63.0 (3)
C3—C4—C5—C6	−12.8 (6)	O5—C23—C24—F4	−71.1 (3)
S1—C4—C5—C6	166.8 (3)	N3—C23—C24—F4	51.9 (4)
O1—C5—C6—C10	−56.5 (4)	C22—C23—C24—F4	168.3 (3)
C4—C5—C6—C10	123.4 (3)	O5—C23—C24—F5	49.6 (4)
O1—C5—C6—C7	65.9 (4)	N3—C23—C24—F5	172.6 (3)
C4—C5—C6—C7	−114.1 (3)	C22—C23—C24—F5	−71.0 (4)
C5—C6—C7—O2	−57.0 (3)	O5—C23—C24—F6	167.8 (3)
C10—C6—C7—O2	65.5 (3)	N3—C23—C24—F6	−69.2 (3)
C5—C6—C7—N1	−179.9 (3)	C22—C23—C24—F6	47.2 (4)
C10—C6—C7—N1	−57.4 (3)	C23—C22—C26—N4	48.8 (3)
C5—C6—C7—C8	63.1 (3)	C21—C22—C26—N4	172.8 (3)
C10—C6—C7—C8	−174.5 (3)	C23—C22—C26—C27	169.7 (2)
O2—C7—C8—F3	−72.8 (4)	C21—C22—C26—C27	−66.3 (3)
N1—C7—C8—F3	50.3 (4)	N4—C26—C27—C28	−136.7 (3)
C6—C7—C8—F3	167.7 (3)	C22—C26—C27—C28	103.7 (4)
O2—C7—C8—F2	48.7 (4)	N4—C26—C27—C32	44.8 (4)
N1—C7—C8—F2	171.8 (3)	C22—C26—C27—C32	−74.8 (4)
C6—C7—C8—F2	−70.8 (4)	C32—C27—C28—C29	1.3 (6)
O2—C7—C8—F1	166.9 (3)	C26—C27—C28—C29	−177.2 (3)
N1—C7—C8—F1	−70.0 (3)	C27—C28—C29—C30	−0.7 (6)
C6—C7—C8—F1	47.3 (3)	C28—C29—C30—C31	0.2 (6)
C5—C6—C10—N2	173.3 (3)	C28—C29—C30—Cl2	−178.4 (3)
C7—C6—C10—N2	48.7 (3)	C29—C30—C31—C32	−0.2 (7)
C5—C6—C10—C11	−66.2 (3)	Cl2—C30—C31—C32	178.4 (3)
C7—C6—C10—C11	169.2 (3)	C30—C31—C32—C27	0.8 (7)
N2—C10—C11—C16	−137.7 (3)	C28—C27—C32—C31	−1.3 (6)
C6—C10—C11—C16	102.4 (4)	C26—C27—C32—C31	177.2 (4)
N2—C10—C11—C12	46.7 (4)	O3—C9—N1—C7	165.8 (4)
C6—C10—C11—C12	−73.2 (4)	N2—C9—N1—C7	−18.2 (5)
C16—C11—C12—C13	1.0 (6)	O2—C7—N1—C9	−77.2 (4)
C10—C11—C12—C13	176.7 (4)	C8—C7—N1—C9	161.8 (3)
C11—C12—C13—C14	−0.9 (7)	C6—C7—N1—C9	43.2 (4)
C12—C13—C14—C15	0.7 (7)	O3—C9—N2—C10	−175.4 (4)
C12—C13—C14—Cl1	179.0 (4)	N1—C9—N2—C10	8.6 (6)
C13—C14—C15—C16	−0.6 (6)	C11—C10—N2—C9	−145.7 (4)
Cl1—C14—C15—C16	−179.0 (3)	C6—C10—N2—C9	−25.5 (5)
C14—C15—C16—C11	0.7 (6)	O6—C25—N3—C23	164.7 (4)
C12—C11—C16—C15	−0.9 (6)	N4—C25—N3—C23	−15.9 (6)
C10—C11—C16—C15	−176.6 (3)	O5—C23—N3—C25	−77.9 (4)
S2—C17—C18—C19	1.3 (7)	C22—C23—N3—C25	43.0 (4)
C17—C18—C19—C20	−0.1 (6)	C24—C23—N3—C25	160.8 (3)
C18—C19—C20—C21	−179.4 (4)	O6—C25—N4—C26	−175.8 (4)
C18—C19—C20—S2	−1.1 (4)	N3—C25—N4—C26	4.8 (7)
C19—C20—C21—O4	165.8 (4)	C27—C26—N4—C25	−142.9 (4)
S2—C20—C21—O4	−12.3 (5)	C22—C26—N4—C25	−22.8 (5)
C19—C20—C21—C22	−14.0 (6)	C2—C1—S1—C4	0.0 (6)
S2—C20—C21—C22	167.8 (2)	C5—C4—S1—C1	−179.0 (4)
O4—C21—C22—C26	−58.9 (4)	C3—C4—S1—C1	0.8 (4)
C20—C21—C22—C26	120.9 (3)	C18—C17—S2—C20	−1.7 (5)
O4—C21—C22—C23	63.1 (4)	C19—C20—S2—C17	1.6 (4)
C20—C21—C22—C23	−117.0 (3)	C21—C20—S2—C17	−179.9 (4)

### Hydrogen-bond geometry (Å, °)

**Table:**

*D*—H···*A*	*D*—H	H···*A*	*D*···*A*	*D*—H···*A*
N1—H1A···F3	0.86	2.38	2.719 (4)	104
N1—H1A···O7^i^	0.86	2.37	3.022 (4)	132
N2—H2A···O7	0.86	2.15	2.914 (4)	147
O2—H2B···O6^ii^	0.89 (4)	1.85 (4)	2.694 (4)	156 (3)
N3—H3A···F4	0.86	2.41	2.733 (4)	103
N3—H3A···O8^iii^	0.86	2.34	3.007 (4)	134
N4—H4A···O8	0.86	2.14	2.921 (4)	151
O5—H5···O3	0.98 (6)	1.72 (6)	2.686 (4)	169 (6)
O7—H7A···O6^iii^	0.84 (7)	2.27 (7)	2.879 (4)	130 (5)
O7—H7B···O4^iv^	0.86 (6)	2.36 (6)	2.996 (5)	131 (5)
O7—H7B···O5^iv^	0.86 (6)	2.18 (6)	2.859 (4)	135 (5)
O8—H8A···O3^v^	0.75 (5)	2.21 (5)	2.897 (4)	153 (4)
O8—H8B···O1^i^	0.83 (5)	2.22 (6)	2.968 (5)	150 (5)
O8—H8B···O2^i^	0.83 (5)	2.27 (6)	2.861 (4)	128 (5)

Symmetry codes: (i) −*x*+1/2, *y*−1/2, *z*; (ii) *x*−1, *y*, *z*; (iii) −*x*+3/2, *y*+1/2, *z*; (iv) −*x*+1/2, *y*+1/2, *z*; (v) −*x*+3/2, *y*−1/2, *z*.
